# Research on the Healing Potential of Rural Community Streets From the Perspective of Audiovisual Integration: A Case Study of Four Rural Communities in China

**DOI:** 10.3389/fpubh.2022.861072

**Published:** 2022-03-11

**Authors:** Erkang Fu, Yuxin Ren, Xi Li, Lei Zhang

**Affiliations:** College of Landscape Architecture, Sichuan Agricultural University, Chengdu, China

**Keywords:** rural community, streetscape, audio-visual interactions, restorative potential, aesthetic preference

## Abstract

Rural communities have become a hot topic in academic circles because of their graceful natural environment and great healing potential. However, existing research still lacks attention to the street space in rural communities and rarely considers its integrated visual and soundscape design in terms of their effect on public health. As a result, the healing potential of rural community streets cannot be fully used in design practice. Relevant audiovisual materials were collected from a field investigation in four rural communities in southwestern China. Based on these data, the subjective and objective healing index data of subjects under comprehensive audiovisual conditions were collected and analyzed through laboratory experiments. The results revealed that type of street space affects healing potential, and the artificial–natural enclosed and natural semi-enclosed streets are the street types with the best healing effect. When the total sound pressure level was 55dB(A), the sound combination with birdsong accounting for 70% had a significant positive effect on improving the healing effect of rural community streets. In contrast, the sound combination with birdsong accounting for 50% or less had no significant effect on improving healing. The subjective healing perception of street space in rural communities was significantly positively correlated with aesthetic preferences. There was also a significant correlation between subjective healing perception and physiological index data in the audiovisual combination. This research explored the impact of different types of street space and sound combinations on the healing effect of rural community streets in an integrated audiovisual environment and provided a scientific basis for the healing landscape design of rural community streets in an integrated audiovisual environment. It was expected to provide new ideas for the construction of rural community landscapes, including acoustic landscapes, to promote physical and mental healing.

## Introduction

As the scale of cities increases, psychological stress and environmental deterioration have seriously threatened urban residents' health. People tend to get close to the natural environment to recover from stress (1, 2). Rural communities' beautiful natural scenery and quiet acoustic environment are the main characteristics of rural communities that distinguish them from built-up urban areas (3). This also results in rural communities having better healing effects than urban environments, making them an ideal place to which urban residents can travel to restore their physical and mental health (4). Streets are the leading basic service facilities for production and life in the vast rural areas. With continuously increasing construction, their status and role in the development of the entire rural community have become increasingly prominent. However, most rural community streets are still dominated by basic functions such as ensuring traffic in the planning and design process, which ignores their healing potential (5). It is worth noting that there are indeed some roads in rural communities that do not match the definition of urban streets in the usual sense (6). According to the actual situation of rural communities, the streets explored in this study refer to open public linear spaces that meet transportation needs, and whose spaces mainly include the space surrounded by the bottom interface and the side interface, and can be perceived as a whole space.

With the emergence of the soundscape concept, the research scope of the acoustic environment has expanded from traditional noise control to multidisciplinary research (7). Related studies have found that the study of the healing potential of the environment by visual perception alone cannot provide comprehensive guidance for actual landscape design (8). Research on integrated audiovisual environments, including soundscapes, could improve the impact of the real environment on people's body and mind (9). Research on the healing potential of rural community street landscapes in an integrated audiovisual environment can provide a scientific basis for the planning and design of rural community street landscapes and meet the aesthetic and healing needs of the public through multisensory experience.

### Street Healing Environment

The theory of healing environment was an important experimental psychology research result from the perspectives of reason and experience (10). Linked with empirical design, attention recovery theory (ART) (11), proposed by Kaplan, provided strong support for the view that the environment has a healing effect. ART argues that the healing environment has four characteristics: being away, fascination, extent, and compatibility, which represent the four-stage gradual influence process of the environment on people's emotional and cognitive recovery (11). Natural landscape elements are considered important indicators for predicting the potential of environmental healing. At present, most research on the healing environment focuses on natural green spaces or green spaces in high-density urban environments (12–14). In recent years, because of their natural characteristics, the streets have received attention from scholars for their potential healing effects. Shao et al. researched urban commuter streets and found that “terrain undulations” and “green plants” could promote subjects' healing experience; elements such as buildings, walls, and cars would hinder subjects from obtaining healing perception to varying degrees (15). Jiang et al. and Xu et al. found a positive correlation between green visibility and healing potential in urban life-type streets through virtual media studies such as images and that the more open the street interface, the more attractive it was (16, 17). Mohamed et al. proved that different kinds of trees near urban streets reduced subjects' negative psychological states of stress, fatigue, confusion and anxiety in different ways (18). These studies have discussed in detail the factors influencing the healing effectiveness of streets in the context of the landscape's visual elements, but most of their research focuses on a single type of street in a high-density urban environment, with less attention paid to the street environments of rural communities, much less to the study of different types of street healing environments in rural communities, making it difficult to summarize the methods for enhancing the healing potential of various types of streets in a targeted manner.

### Audiovisual Integrated

Research on the healing environment from the perspective of visual or auditory perception has found that natural visual features (such as water, trees, flowers) and natural auditory features (such as birdsong and the sound of running water) has a positive predictive effect on the healing potential of the environment (9, 19). However, the environment is an ecological entity that includes landscapes, soundscapes and human (20), and its effects on human health and wellbeing also accumulate through a variety of sensory pathways. In particular, the interaction between vision and hearing has a significant impact on the healing potential of the environment. Previous studies have found that visual stimuli affect the comfort and preference evaluation of soundscapes, and vice versa (21, 22). For example, the evaluation of soundscape with visual landscape is significantly higher, audiovisual stimuli associated with nature are thought to have better health benefits than visual stimuli alone (9), and natural landscape elements can improve the quality of residential restoration and reduce traffic-related annoyances by visually identifying and screening noise sources (23, 24).

Sound recognition and classification has become indispensable to research on the integrated audiovisual environment, and its impact on the healing potential of the environment cannot be ignored. In natural parks, traffic noise had a negative impact on the beauty, preference, naturalness, and security of the scenery (25), whereas birdsong was considered the natural sound that most enhanced environmental aesthetic preference and healing perception (26). The interaction between traffic noise and birdsong has also attracted academic attention. Birdsong has been proved an effective way to improve soundscape quality, especially in reducing noise damage to health (7). In addition to the type of sound, loudness is also an important predictor of restorability. Sound pressure level (SPL) is a common physical quantity for sound loudness measurement (27), with a direct effect on noise-induced annoyance; and within a certain SPL range, different sound types can play a certain role in intervention. For example, when traffic noise increases, louder birdsong can effectively improve soundscape quality, but when the traffic noise exceeds 57.5dB, annoyance increases as birdsong SPL increases (7). The interaction between the sound type and the SPL has been shown to have a certain impact on the environment's healing potential, but existing research lacks further discussion on this aspect.

### Research Questions

In addition, controversies remain in the current healing environment research based on the integrated audiovisual environment. For example, some studies have pointed out that a higher green viewing rate causes people to overestimate the perceived loudness of noise (28). This is contrary to the conclusion, in a large number of published research results, that greening can alleviate noise annoyance (29). This may be because experiments in real environments are affected by many uncontrollable factors; the use of controlled laboratory experiments can provide a realistic and immersive audiovisual reproduction system to avoid such problems. The reproduction method based on audio external playback and virtual image is more realistic and reliable for overall soundscape quality, sound-related attributes and environment-related spatial attributes. Therefore, this research has been conducted in the laboratory to improve sound type and SPL processing precision and reduce the influence of other factors.

In view of the above discussion, the main research questions of this study are as follows:

(1) What are the differences in the subjective perceptual evaluations of different types of rural community streets?
(2) How does the interaction between sound type and sound pressure level affect the subjective perception evaluation of rural community street environments?
(3) What is the relationship between visual aesthetic preference, auditory subjective loudness and subjective perception of healing in rural community street space in an integrated audiovisual environment?
(4) What is the correlation between subjective healing perceptions and objective physiological data?

## Materials and Methods

### Study Area and Photographs

**Figure 1:**
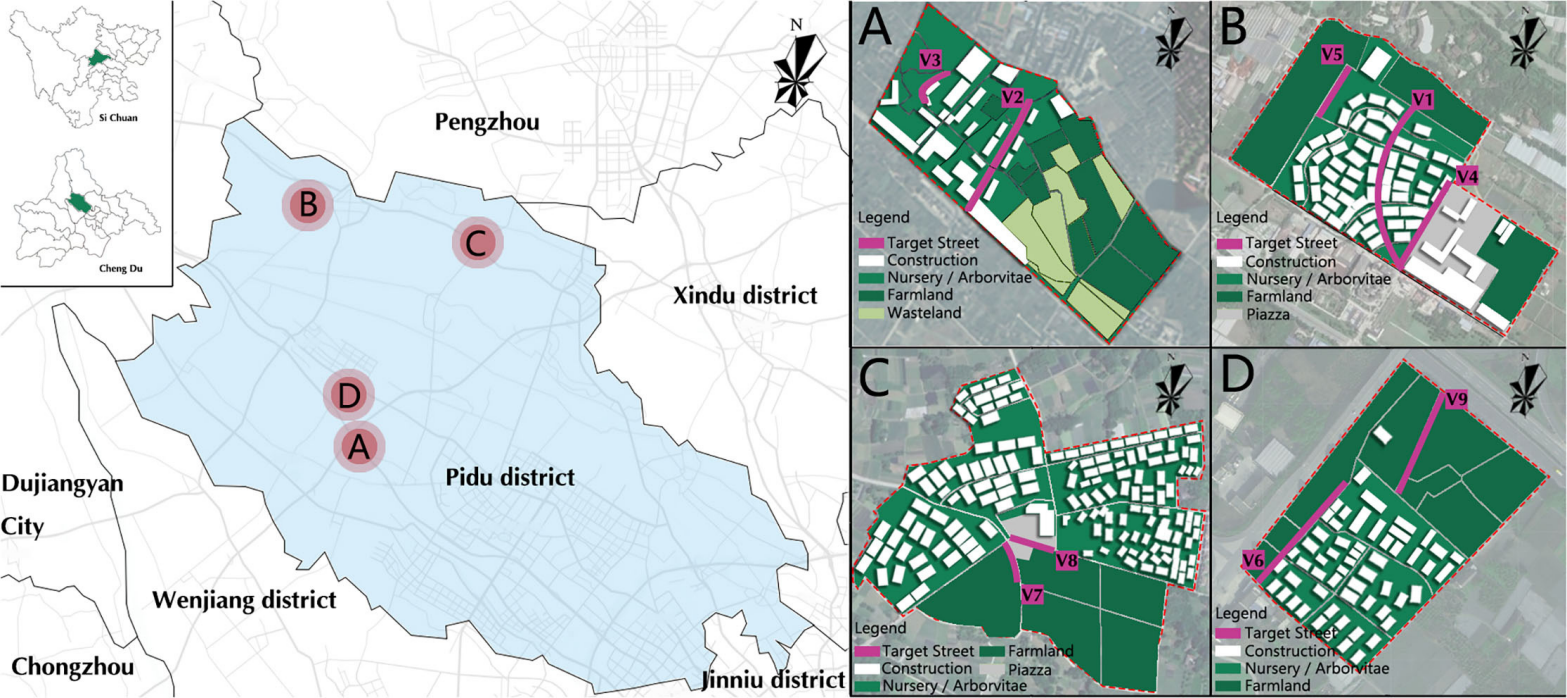
Study area and the location of nine different types of streets.

Street pictures have the advantages of convenient operation and strong experimental control. It has been proved that there is no significant difference from the experimental data when viewing the actual landscape (30–32). The photographs used in this study were taken in four rural communities in the Pidu District of Chengdu, Sichuan Province, China (Figure 1). After field investigation, the rural street environment was divided into nine types according to spatial openness and street interface elements by five experienced landscape architects. These include artificial enclosure, natural enclosure, artificial–natural enclosure, artificial semi-enclosed, natural semi-enclosed, artificial–natural semi-enclosure, natural–artificial semi-enclosure, artificial open, and naturally open type. Artificial enclosure, artificial semi-enclosed, and artificial open were the street landscape types with a higher degree of manpower in rural communities. The two sides of their interface were composed of buildings, walls, or hard pavement, but the height of the buildings on both sides of the street in the rural community is about 3–8 m, much lower than that of the urban buildings, and the building density was also lower.

Artificial–natural enclosure was a type of landscape with a moderate degree of artificiality. One side of the street is surrounded by buildings or walls, and the other side is surrounded by trees. Artificial–natural semi-enclosure and natural–artificial semi-enclosure type referred to one side of the street surrounded by buildings or trees, and the other side was open farmland or square. Natural enclosure, natural semi-enclosure and naturally open had the lowest degree of manpower. Compared with urban streets, farmland was a unique interface element of rural community streets.

**Figure 2:**
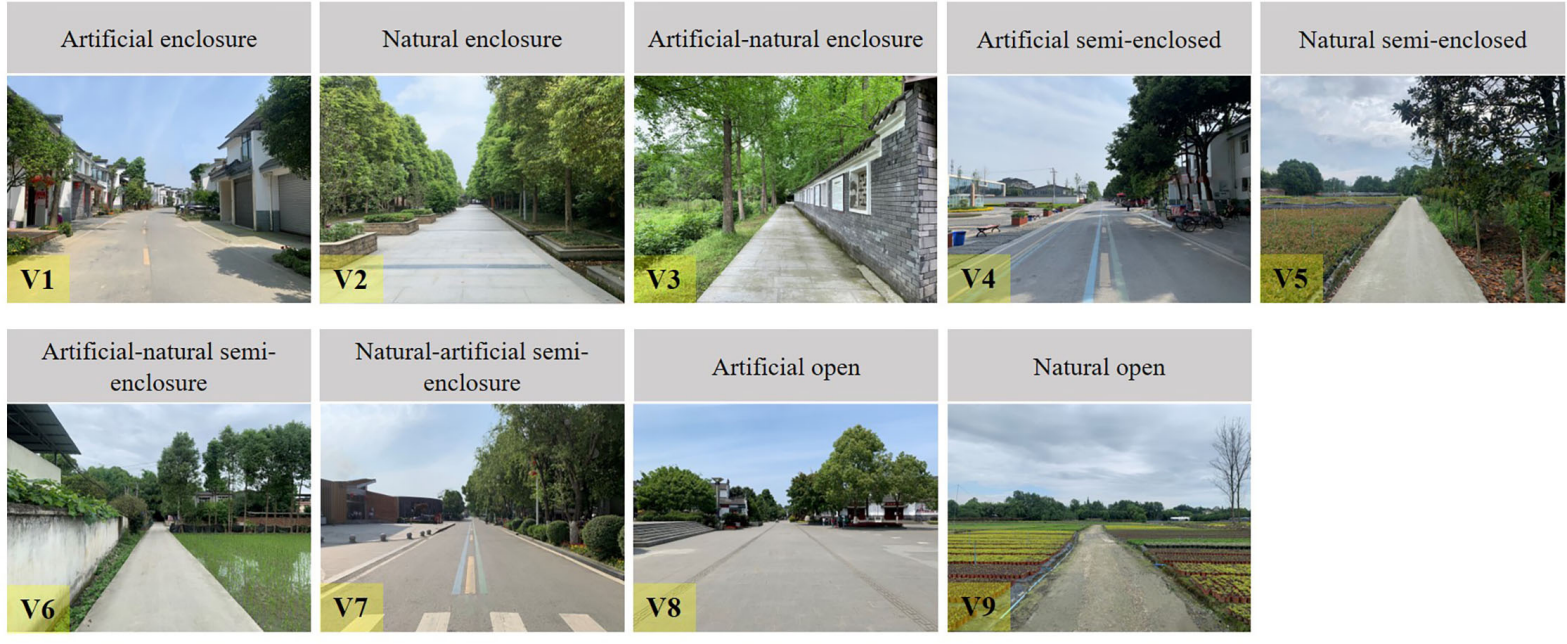
Pictures of the different street types taken in study area.

According to the above classification, nine rural community streets in the study area were selected as the research objects, and field photos were taken in May 2021 when the weather was clear and haze free (PM2.5 < 100). Shooting took place in the morning and afternoon, between 9:00 and 11:30 and between 13:00 and 16:00. The Nikon D7500 SLR camera was used to shoot street photographs. The camera was placed on a stand with a height of 1.5 m. The shooting angle was parallel to the road surface, and the position was centered on the sidewalk. The shooting maintained a horizontal frame and a certain depth of field to capture the main features of a given scene. A total of 312 photographs were taken. After excluding those that did not meet the requirements, 119 candidate street photographs remained, including 15 sheets of artificial enclosure streets, 15 sheets of natural enclosure streets, 12 sheets of artificial–natural enclosure streets, 13 sheets of artificial–natural semi-enclosure streets, 17 sheets of natural semi-enclosure, 15 sheets of artificial–natural semi-enclosure streets, 12 sheets of natural–artificial semi-enclosure streets, 10 sheets of artificial open streets, and 10 sheets of naturally open streets. These photos were imported into Adobe Photoshop 2019CC software to remove the text, symbols, and other irrelevant factor in the photographs. The brightness, color saturation, and contrast of each photograph were adjusted for consistency to eliminate the perceptual deviation caused by the photographs itself. A representative photograph of each landscape type was selected by five landscape architects. Their criteria included good photographic quality and wide differences between different types of street space. Finally, nine photographs were selected as experimental materials. each of which represents one rural community streets type (Figure 2). Because this research aimed to explore the differences in the healing potential of different types of rural community streets at the visual level, the street photographs with the most typical characteristics were selected rather than the strongest or most attractive landscape photographs.

### Sound Collection and Combination of Audiovisual Scenes

In this study, birdsong and traffic noise were recorded by field investigation. Mono source audio was recorded near the source without interference from other sounds. The recording were placed on a tripod 1.5 m above the ground and more than 3.5 m from other reflectors. No rain, snow or thunder and lightning occurred during the measurement and acquisition period, and the wind speed was <5 m/s. Although an analysis based on sound level measurements was not the main objective of this research, sound levels (dBA) were also recorded in order to obtain a guidance of the physical sound levels at the rural community streets. To accomplish this, a total of 9 sampling points were set up, all located within 5 m of the research objects, and the measurement time for each sampling point was 10 min (33). The average of the L_Aeq_ values measured at 9 sampling points was used as the final rural community street environmental SPL, and the L_Aeq_ value is 55dB(A). The two audio clips with the best recording effects were imported into Adobe Audition CC2017 software and processed into clear and complete audio with the same frequency. Finally, the two sound audios were combined according to different sound pressure level ratios, and three composite sound audios composed of two sound types and three SPL were interactively combined (Table 1) for 30 s each (34). The SPL calibration of the above audio was done using Zhaohua Electronics CRY318 artificial ear wearing SONY 1000XM4 noise reduction headphones. During the calibration process, the volume was adjusted using Adobe Premiere so that all sound signals were close to 55.5dB(A).

**Table 1:** Sound combinations.

**Combinations**	**Birdsong**	**Traffic noise**
S0	None	None
S1	70%	30%
S2	50%	50%
S3	30%	70%

The nine visual images and four composite sound combinations extracted by the survey were combined one by one in Adobe Premiere software, and finally 36 sets of audiovisual scene videos were obtained as control experimental materials. The duration of each set of videos was 30 s.

### Participants

Luo et al. used college students as research objects in the research on the perception and restoration of rural blue-green space and obtained good results (35). Therefore, college students who had been under chronic stress and sub-health conditions for a long time were selected as the research objects of this study. Posters on campus were used to recruit students meeting the following requirements as participants in this study: (1) between the ages of 19 and 26; (2) in good physical and mental health, without alcoholism, smoking, or other bad habits; (3) no major recent life changes; and (4) normal vision and hearing.

Studies have found that people are more likely to experience healing when they are alone (36). Participants in this experiment were asked to participate one by one, and there was no external interference during the experiment. Fifty-seven people were recruited for this experiment, including 28 males and 29 females, with an average age of 22.6 ± 1.7 years old. During the entire study period, participants were asked to avoid drinking and vigorous physical activity. The experiment was completed in approximately 40 min. The study was approved by the local ethics committee of the College of Landscape Architecture, Sichuan Agricultural University, China; all subjects voluntarily participated in the experiment.

### Measurement of Participants' Perception

#### Subjective Evaluation Questionnaire

The subjective evaluation questionnaire of this study consisted of three parts: the visual aesthetic quality (VAQ) of visual perception of landscape, the tranquility rate (TR) in auditory perception, and the restorative components scale (RCS). VAQ, defined as “beautiful and striking” (37), was rated using a 5-point Likert scale (ranging from 0, “totally disagree,” to 4, “totally agree”). “Quiet” and “Noisy” were used to describe the two ends of the tranquility rate (ranging from 0, “very noisy,” to 4, “very quiet”) (38). The RCS developed by Laumann et al., used as an evaluation tool for subjective healing perception in this study (39), consisted of four dimensions: being away, fascination, extent, and compatibility. Four items suitable for street environmental assessment were selected as a short modified questionnaire (40) (Table 2), and subjects used 0–4 points to describe the degree of agreement between each question and their actual perception. The score of each dimension was obtained by calculating the average score of 57 subjects, and the average of the overall scores of all dimensions was taken as the overall healing perception score of each audiovisual scene.

**Table 2:** The restorative components scale used in this experiment.

**Dimension**	**Description**	**Scales**
Being-away (B)	B1 There allows me to temporarily forget the troubles of work and daily life.	0 1 2 3 4
Extent (E)	E1 There are many beautiful associations here.	0 1 2 3 4
Fascination (F)	F1 There's a lot here that appeals to me.	0 1 2 3 4
Compatibility (C)	C1 There gives me the opportunity to do what I love to do.	0 1 2 3 4

#### Physiological Indicators

Pulse and blood pressure measurement with the OMRON HEM-7124 portable blood pressure meter (41). When the human body was in a relaxed state, diastolic and systolic blood pressure decrease, whereas in a high-pressure and tense state, diastolic and systolic blood pressure increase. At rest or relaxation, the parasympathetic nervous system becomes more functional, pulse becomes slower, and blood output at each stroke decreases. When the individual is in a state of high tension or stress response, sympathetic nervous system excitation increases, parasympathetic nervous system excitation decreases, and the body shows a rapid pulse.

### Procedure

**Figure 3:**
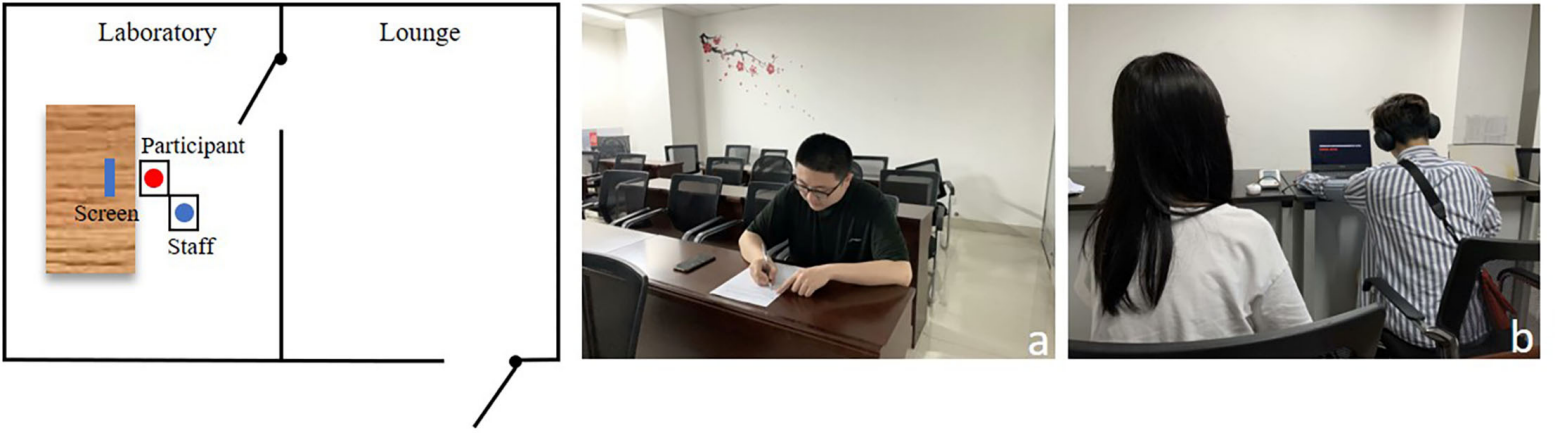
Experimental scene [**(a)** the participants was waiting in the waiting room to rest and fill in relevant information; **(b)** the experimenter is conducting the experiment].

The experiment was carried out in a laboratory that was ventilated, quiet, and soundproof with no visual disturbance (Figure 3). In addition to the participants, a staff member was present in the laboratory. The staff did not need to communicate with the participants during the experiment. Images and audio and informative information were presented in the slideshow.

**Figure 4:**
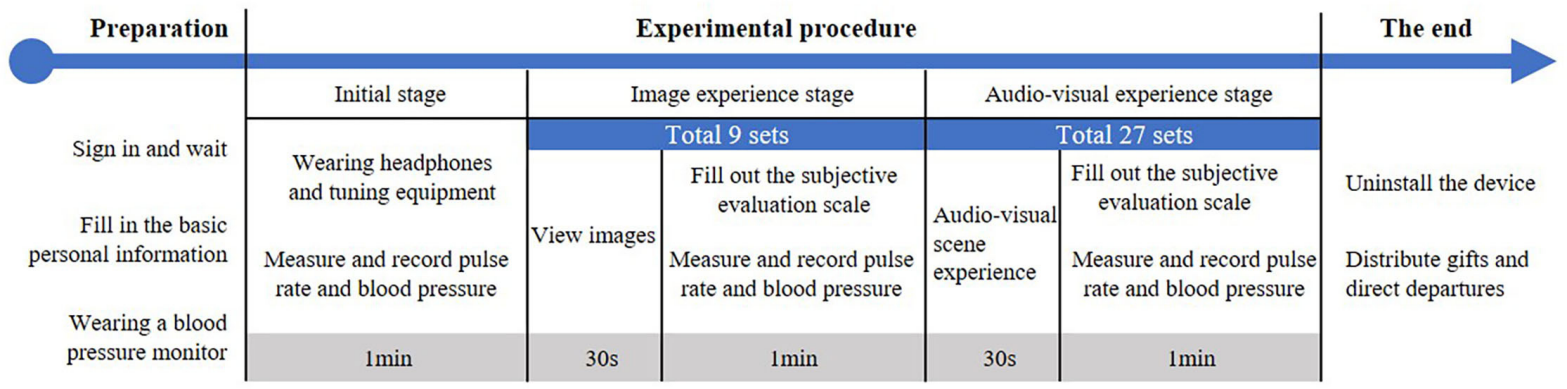
Flow chart of single-person experiment for visual landscape and auditory soundscape evaluation.

The experiment was carried out May 22–27, 2021. The daily average temperature and humidity during the experiment were 24.3 ± 2.3°C, 76.2 ± 9.9%. The participants were randomly divided into groups of ten. Participants received an email 1 day before the experiment containing experimental precautions and process details. This helped participants to complete the experiment smoothly and reduced field data collection errors. On the day of the experiment, the participants arrived in the experimental lounge to sit and wait for 10 min and fill in the written informed consent form and personal information. Afterward, the participants were asked to sit in front of a blank computer screen in the laboratory and wear noise-canceling headphones and a portable blood pressure monitor, and the staff measured the participants' initial heart rate and blood pressure. During the experiment, the participants first watched nine silent photographs and then experienced 27 groups of audiovisual scenes with added sound combinations. All scenes were presented in random order. Each photographs and scene were displayed continuously for 30 s (30), and subsequent scenes were displayed after an interval of 1 min. During the interval, the participants were asked to rate the VAQ, tranquility rating (TR) and RCS of the previous scene, and the staff measured the participants' heart rate and blood pressure and recorded them (Figure 4).

### Data Analysis

SPSS 22.0 was used to process the obtained data in this study. Reliability analysis was used to test the internal consistency reliability of the data from the 57 subjective evaluation questionnaires obtained in the experiment. Multivariate ANOVA was used to analyze the differences of subjective evaluation results under different street types and sound combinations in rural communities. The Kruskal-Wallis test was used to analyze the effect of street type and sound combination on the participants' physiological indicators. Finally, Pearson correlation was used to explore the correlation between VAQ, TR, RCS, and physiological index data of street space in rural communities under audiovisual interaction.

## Results

### Internal Consistency Reliability

Cronbach's alpha value was used to measure the internal consistency of all subjective evaluation scales in the experiment. It is generally believed that Cronbach's alpha value >0.801 represents good consistency within the data (32). Between-group reliability test analysis showed that the RCS under visual photographs stimulation and the RCS under combined audiovisual stimulation had high internal consistency. Specifically, the Cronbach's alpha value of the questionnaire evaluated under the visual image stimulus was 0.925, and the Cronbach's alpha value of the questionnaire was evaluated under the audiovisual combination stimulus of 0.913.

### Influence of Street Type and Sound Combination on the Subjective Perception Evaluation of Street Space in Rural Communities

#### Difference Analysis of Street Type and Sound Combination on the VAQ

Multivariate ANOVA showed that different street types and sound combinations had significant effects on the VAQ of street space in rural communities, and there was no interaction between street types and sound combinations (*p* = 0.96 > 0.05).

**Figure 5:**
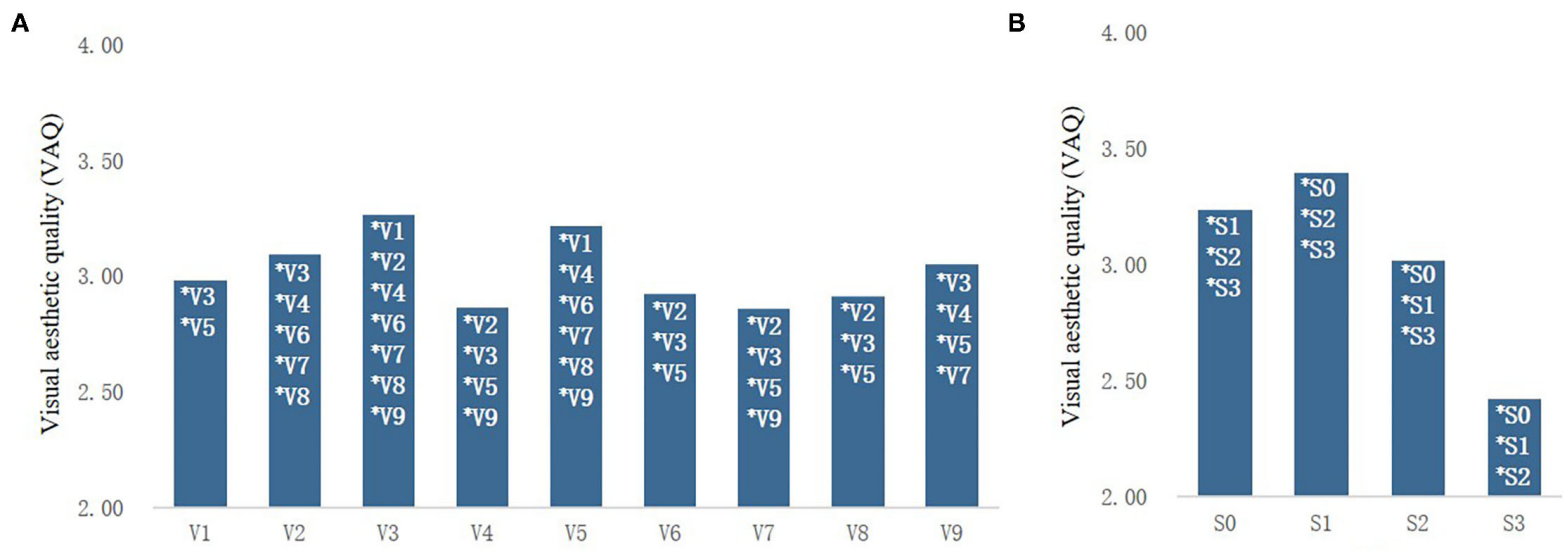
Comparison of visual aesthetic quality (VAQ) for different street types **(A)** and different sound combinations **(B)**.

As shown in Figure 5, the average score of VAQ of all types of streets in rural communities was super-moderate (>2 points), indicating that the overall landscape condition of rural community streets was good, but there were significant differences in scores among different types of streets. Specifically, the VAQ score of artificial–natural enclosed (V3) streets was the highest (3.26 ± 0.84), which was significantly higher than that of all types of streets except natural semi-enclosed (V5) streets. Followed by natural semi-enclosed (V5) streets, its score was significantly higher than other types of streets (3.22 ± 1.08) except for that of artificial–natural enclosed (V3) streets and natural enclosed (V2) streets. Artificial semi-enclosed (V4) streets (2.86 ± 0.98) and artificial–natural semi-enclosed (V6) streets (2.86 ± 0.93) had the lowest VAQ evaluation, significantly lower than natural enclosed (V2) streets, artificial–natural enclosed (V3) streets, natural semi-enclosed (V5) streets, and natural open (V9) streets.

And the overall VAQ evaluation (3.39 ± 0.68) of the street images with the S1 sound combination was significantly higher than that with silent images only (3.24 ± 0.78), whereas the score of street images with S2 sound combination was significantly lower than that with silent images only (3.02 ± 0.77). Street images with S3 sound combination scored the lowest (2.42 ± 1.17). Results revealed that the VAQ evaluation of street space in rural communities was significantly improved by the sound combination with 70% birdsong but significantly decreased in the sound combination with 50% or less birdsong.

#### Difference Analysis of Street Type and Sound Combination on Auditory Subjective Loudness Evaluation

Multivariate ANOVA was used to explore the influencing factors of TR of street photographs in rural communities. The results showed that both street type and sound combination had significant influence on the TR of rural communities, and there was an interactive relationship between street type and sound combination.

**Figure 6:**
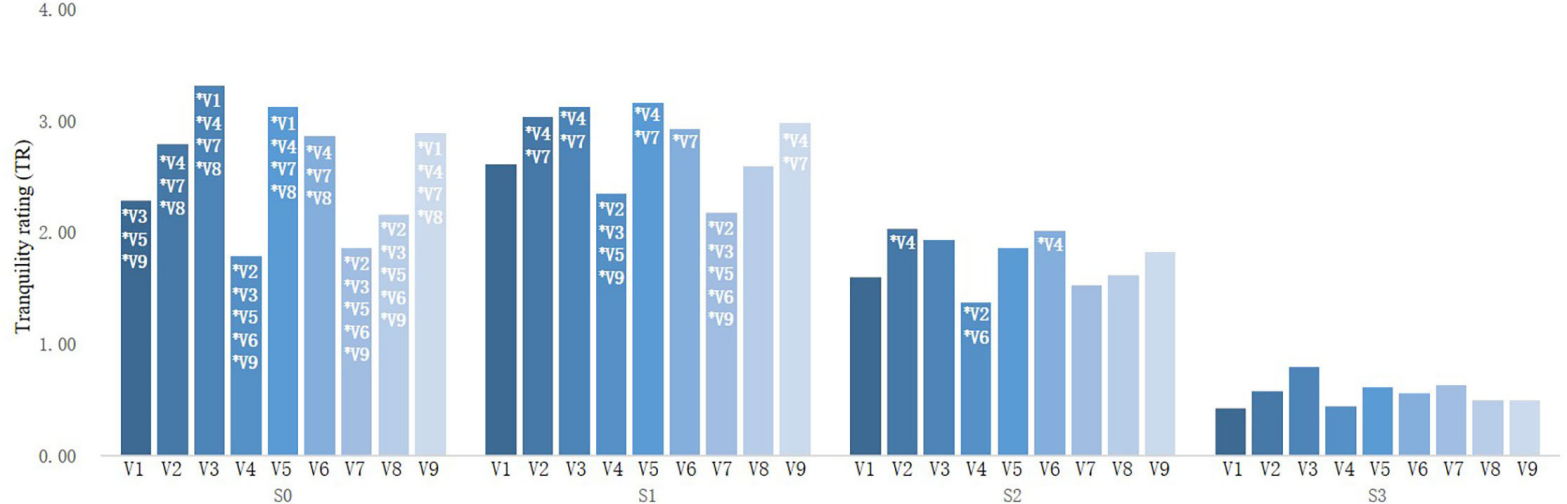
Comparison of different types of street TR.

Simple effect analysis was used to explore the interaction between street types and sound combinations. It was found that the participants' TR for artificial–natural enclosure (V3) streets was the highest (3.32 ± 0.82) and significantly higher than that of artificial enclosure (V1) streets, artificial semi-enclosed (V4) streets, artificial–natural semi-enclosure (V7) streets, and artificial open (V8) streets when viewing photographs without sound. The TR of artificial semi-enclosed (V4) streets was the lowest (1.79 ± 1.25) and was significantly lower than that of natural enclosure (V2) streets, artificial–natural enclosure (V3) streets, natural semi-enclosed (V5) streets, natural–artificial semi-enclosure (V6) streets, and naturally open (V9) streets. After S1 was added, the TR of natural semi-enclosed (V5) streets was the highest (3.16 ± 0.85), but it was significantly higher only than that of artificial semi-enclosed (V4) streets and artificial-natural semi-enclosure (V7) streets, whereas the TR of artificial-natural semi-enclosure (V7) streets was the lowest (2.18 ± 1.09), significantly lower than that of natural enclosure (V2) streets, artificial–natural enclosure (V3) streets, natural semi-enclosed (V5) streets, natural–artificial semi-enclosure (V6) streets, and naturally open (V9) streets. After S2 was added, the TR of natural enclosure (V2) streets was the highest (2.04 ± 1.24), significantly higher than that of artificial semi-enclosed (V4) streets (1.37 ± 0.98). In the context of S3 sound combination, street type had no significant effect on TR of street space in rural communities (Figure 6).

**Figure 7:**
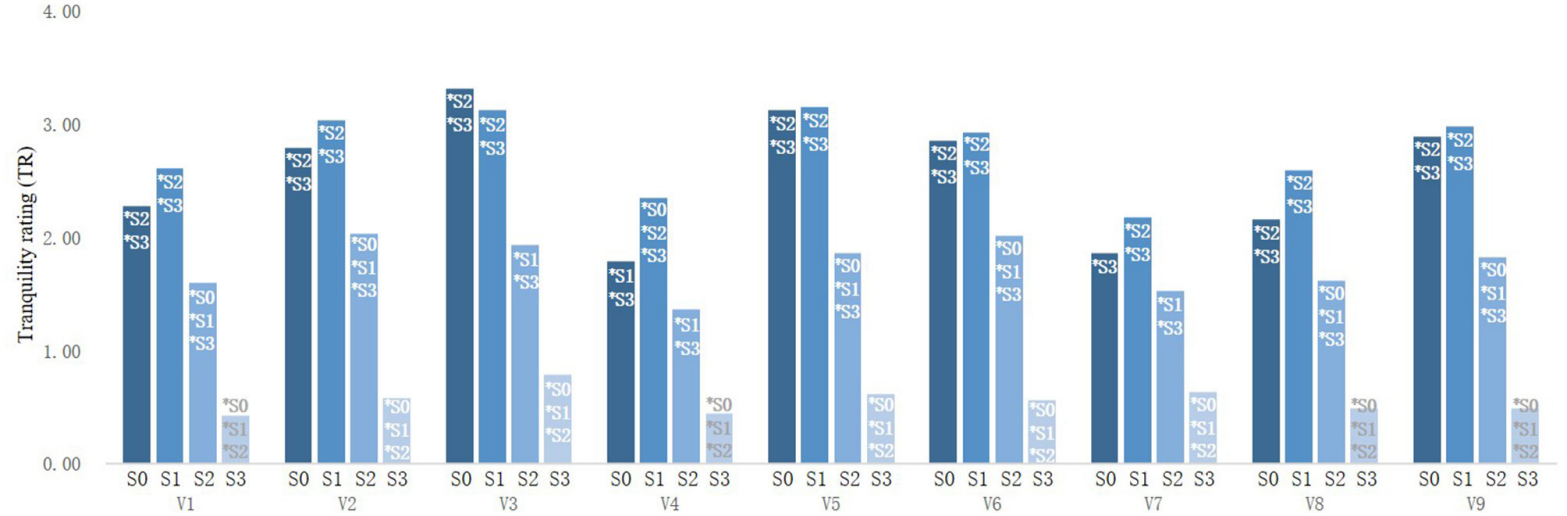
Comparison of street TR under different sound combinations.

For street types other than artificial semi-enclosed (V4) streets, there was no significant difference in TR when viewing images without sound and adding S1 sound combination (Figure 7). All types of street photographs combined with S1 had significantly higher TR than those combined with S2 or S3. The TR of all types of street photographs combined with S2 were significantly higher than those of S3. In conclusion, the sound combination with 70% bird song (S1) improved the TR of street space in rural communities more than other sound combinations (S2 and S3) and brought people more peaceful auditory experiences.

#### Effects of Street Type and Sound Combination on Subjective Healing Perception

Multivariate ANOVA was used to analyze whether different types of streets and different sound combinations differed significantly in subjective healing perception of street space in rural communities. It was found that different types of streets and different sound combinations had significant effects on the evaluation of RCS of street space in rural communities and that there was no interaction between street types and sound combinations.

**Figure 8:**
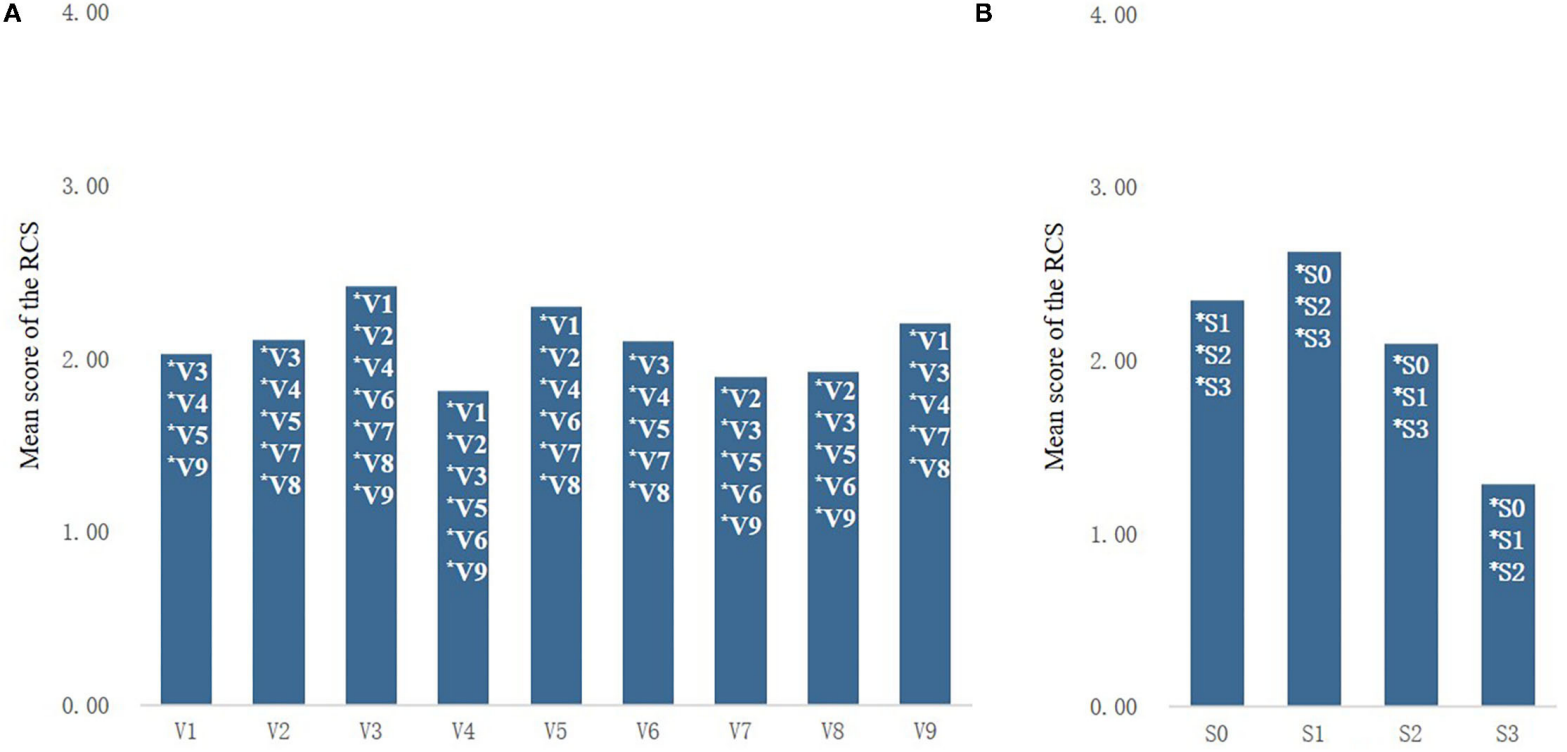
Comparison of RCS scores for different street types **(A)** and different sound combinations **(B)**.

The questionnaire results (Figure 8) showed that the RCS score of artificially enclosed (V3) streets was the highest (2.42 ± 0.88), which was significantly higher than that of all types of streets except naturally enclosed (V5) streets. The score for natural semi-enclosed (V5) streets was 2.30 ± 0.91, significantly higher than that of other types except for artificial–natural enclosed (V3) streets. The lowest RCS score was found in the artificial semi-enclosure (V4) streets (1.81 ± 0.98).

The RCS evaluation of the street photographs with the S1 (2.26 ± 0.69) was significantly higher than the score when only watching the photographs without sound (2.35 ± 0.83), whereas the street photographs with the S2 were significantly lower than the score when viewing the silent photographs was (2.10 ± 0.80), and the street image that added the S3 sound combination had the lowest score (1.28 ± 0.92). The results showed that the sound combination with 70% birdsong (S1) could significantly improve the subjective healing perception of the street space in rural communities, whereas the sound combination with 50% birdsong or less (S2 and S3) had a significant negative impact on the subjective healing perception of the street space in rural communities.

### Effects of Street Type and Sound Combination on Objective Physiological Indicators

**Figure 9:**
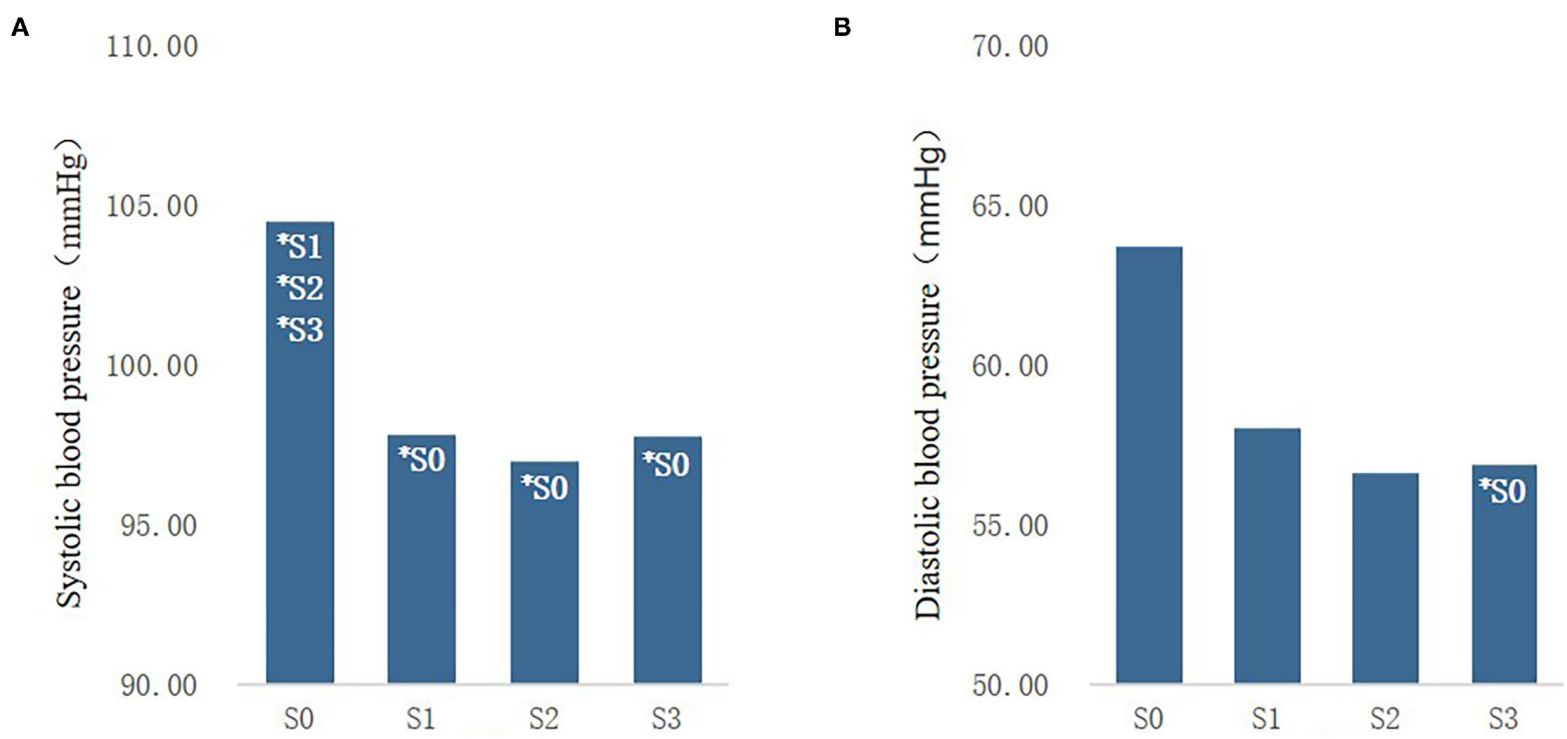
Comparison of systolic blood pressure **(A)** and diastolic blood pressure **(B)** under different sound combinations.

The Kruskal-Wallis test showed that neither the street type nor the sound combination in the rural community had a significant effect on the participants' pulse, whereas the sound combination had a significant effect on the participants' systolic and diastolic blood pressure (Figure 9). The data demonstrated that no matter what sound combination was added, the participants' systolic and diastolic blood pressure were significantly lower than those who only watched the silent photographs, but no significant between-group differences were found for the three sound combinations.

### Visual Aesthetic Quality (VAQ) and Subjective Healing Perception

The results of Pearson correlation showed that when viewing the artificial–natural enclosed (V3) street photograph Under silent conditions, there was no correlation between the VAQ and the scores of “fascination” and “compatibility” factors. After the addition of S3 sound combination, the VAQ of natural semi-enclosed (V5) streets was not correlated with the evaluation of the four healing factors. In the rest of the audiovisual combinations, there was a significant positive correlation between aesthetic preference and various factors of RCS (Table 3). Overall, for all audiovisual scenes, there was a significant positive correlation between VAQ and total score of RCS. It showed that the VAQ of rural community streets was a positive predictor of subjective healing perception.

**Table 3:** Pearson's correlation results between VAQ and evaluation of RCS.

**Sound combination**	**Street type**	**Being away**	**Extent**	**Fascination**	**Compatibility**	**Total score of RCS**
S0	V1	0.605^**^	0.435^**^	0.588^**^	0.665^**^	0.665^**^
	V2	0.444^**^	0.515^**^	0.441^**^	0.436^**^	0.552^**^
	V3	0.537^**^	0.298^*^	0.216	0.210	0.441^**^
	V4	0.641^**^	0.562^**^	0.504^**^	0.593^**^	0.695^**^
	V5	0.600^**^	0.521^**^	0.470^**^	0.536^**^	0.655^**^
	V6	0.538^**^	0.411^**^	0.367^**^	0.287^**^	0.477^**^
	V7	0.445^**^	0.480^**^	0.378^**^	0.467^**^	0.557^**^
	V8	0.552^**^	0.581^**^	0.529^**^	0.591^**^	0.703^**^
	V9	0.633^**^	0.615^**^	0.473^**^	0.537^**^	0.673^**^
S1	V1	0.693^**^	0.596^**^	0.454^**^	0.499^**^	0.669^**^
	V2	0.509^**^	0.491^**^	0.464^**^	0.423^**^	0.600^**^
	V3	0.687^**^	0.571^**^	0.458^**^	0.502^**^	0.675^**^
	V4	0.588^**^	0.567^**^	0.457^**^	0.510^**^	0.628^**^
	V5	0.596^**^	0.538^**^	0.492^**^	0.474^**^	0.623^**^
	V6	0.545^**^	0.528^**^	0.461^**^	0.523^**^	0.626^**^
	V7	0.601^**^	0.619^**^	0.507^**^	0.562^**^	0.683^**^
	V8	0.530^**^	0.441^**^	0.390^**^	0.490^**^	0.584^**^
	V9	0.596^**^	0.538^**^	0.523^**^	0.526^**^	0.652^**^
S2	V1	0.542^**^	0.555^**^	0.439^**^	0.615^**^	0.638^**^
	V2	0.543^**^	0.569^**^	0.519^**^	0.591^**^	0.658^**^
	V3	0.549^**^	0.429^**^	0.359^**^	0.431^**^	0.551^**^
	V4	0.583^**^	0.513^**^	0.487^**^	0.510^**^	0.630^**^
	V5	0.556^**^	0.563^**^	0.430^**^	0.561^**^	0.642^**^
	V6	0.604^**^	0.570^**^	0.403^**^	0.491^**^	0.627^**^
	V7	0.399^**^	0.454^**^	0.362^**^	0.493^**^	0.518^**^
	V8	0.613^**^	0.564^**^	0.514^**^	0.502^**^	0.678^**^
	V9	0.596^**^	0.581^**^	0.457^**^	0.483^**^	0.629^**^
S3	V1	0.717^**^	0.652^**^	0.633^**^	0.682^**^	0.761^**^
	V2	0.646^**^	0.660^**^	0.562^**^	0.620^**^	0.709^**^
	V3	0.618^**^	0.655^**^	0.488^**^	0.588^**^	0.673^**^
	V4	0.647^**^	0.660^**^	0.589^**^	0.593^**^	0.690^**^
	V5	0.165	0.205	0.160	0.212	0.224^*^
	V6	0.687^**^	0.670^**^	0.574^**^	0.615^**^	0.722^**^
	V7	0.646^**^	0.675^**^	0.589^**^	0.662^**^	0.718^**^
	V8	0.618^**^	0.678^**^	0.646^**^	0.573^**^	0.696^**^
	V9	0.700^**^	0.698^**^	0.568^**^	0.572^**^	0.739^**^

* *P < 0.05*

** *P < 0.01*.

### Tranquility Rating (TR) and Evaluation of RCS

When viewing the silent artificial–natural enclosed street (V3) photograph, there was no correlation between TR and “Fascination.” But in other audiovisual combinations, there was a significant positive correlation between TR and various factors of RCS (Table 4). The TR of all audiovisual combinations was significantly positively correlated with the total score of RCS. Therefore, the TR of street space in rural communities was also an important predictor of subjective healing perception.

**Table 4:** Pearson's correlation results between TR and evaluation of RCS.

**Sound combination**	**Street type**	**Being away**	**Extent**	**Fascination**	**Compatibility**	**Total score of RCS**
S0	V1	0.756^**^	0.565^**^	0.588^**^	0.556^**^	0.729^**^
	V2	0.699^**^	0.565^**^	0.457^**^	0.530^**^	0.683^**^
	V3	0.570^**^	0.471^**^	0.260	0.271^*^	0.551^**^
	V4	0.660^**^	0.599^**^	0.438^**^	0.445^**^	0.653^**^
	V5	0.686^**^	0.497^**^	0.484^**^	0.426^**^	0.646^**^
	V6	0.721^**^	0.481^**^	0.456^**^	0.360^**^	0.600^**^
	V7	0.641^**^	0.468^**^	0.261^*^	0.325^**^	0.539^**^
	V8	0.683^**^	0.496^**^	0.275^*^	0.296^**^	0.556^**^
	V9	0.712^**^	0.558^**^	0.498^**^	0.513^**^	0.681^**^
S1	V1	0.602^**^	0.506^**^	0.451^**^	0.421^**^	0.593^**^
	V2	0.694^**^	0.559^**^	0.482^**^	0.349^**^	0.661^**^
	V3	0.692^**^	0.596^**^	0.548^**^	0.516^**^	0.718^**^
	V4	0.596^**^	0.577^**^	0.452^**^	0.454^**^	0.617^**^
	V5	0.696^**^	0.634^**^	0.584^**^	0.517^**^	0.723^**^
	V6	0.717^**^	0.595^**^	0.548^**^	0.495^**^	0.720^**^
	V7	0.679^**^	0.587^**^	0.462^**^	0.451^**^	0.652^**^
	V8	0.644^**^	0.465^**^	0.432^**^	0.434^**^	0.625^**^
	V9	0.759^**^	0.573^**^	0.535^**^	0.483^**^	0.705^**^
S2	V1	0.618^**^	0.562^**^	0.462^**^	0.525^**^	0.645^**^
	V2	0.627^**^	0.628^**^	0.532^**^	0.481^**^	0.674^**^
	V3	0.597^**^	0.485^**^	0.403^**^	0.506^**^	0.625^**^
	V4	0.700^**^	0.609^**^	0.494^**^	0.509^**^	0.699^**^
	V5	0.626^**^	0.586^**^	0.412^**^	0.490^**^	0.647^**^
	V6	0.736^**^	0.686^**^	0.511^**^	0.457^**^	0.727^**^
	V7	0.606^**^	0.618^**^	0.447^**^	0.536^**^	0.670^**^
	V8	0.641^**^	0.565^**^	0.538^**^	0.414^**^	0.671^**^
	V9	0.543^**^	0.546^**^	0.490^**^	0.478^**^	0.613^**^
S3	V1	0.649^**^	0.610^**^	0.555^**^	0.580^**^	0.680^**^
	V2	0.676^**^	0.652^**^	0.547^**^	0.646^**^	0.721^**^
	V3	0.590^**^	0.543^**^	0.429^**^	0.539^**^	0.605^**^
	V4	0.712^**^	0.702^**^	0.549^**^	0.576^**^	0.706^**^
	V5	0.430^**^	0.545^**^	0.511^**^	0.472^**^	0.590^**^
	V6	0.664^**^	0.504^**^	0.484^**^	0.561^**^	0.629^**^
	V7	0.720^**^	0.653^**^	0.515^**^	0.569^**^	0.689^**^
	V8	0.648^**^	0.664^**^	0.564^**^	0.518^**^	0.664^**^
	V9	0.667^**^	0.535^**^	0.538^**^	0.574^**^	0.676^**^

* *P < 0.05*

** *P < 0.01*.

### Subjective Healing Perception and Physiological Indicators

Based on the above results, it was found that the sound combination had a significant effect on the participants' physiological indicators. Canonical correlation analysis was used to explore whether there was a relationship between subjective healing perception and physiological indicators under the influence of different sound combinations. The results showed that there was a significant correlation between evaluation of RCS and physiological index data in the street audiovisual combinations except for the silent street photographs. When the street photographs were combined with S1, the higher the evaluation of the four factors in RCS, the smaller the participants' pulse rate and the higher the systolic and diastolic blood pressure. When street photographs were combined with S2, with the increase in the evaluation of the four factors in RCS, participants' pulse rate and systolic blood pressure decreased and diastolic blood pressure increased. In the audiovisual combination of street photographs combined with S3, participants' pulse rate and diastolic blood pressure decreased and systolic blood pressure increased with the increase of “extent” and “compatibility” factor evaluation scores. The opposite was true when “being away” and “fascination” increased (Table 5).

**Table 5:** Results of canonical correlation analysis between RCS evaluation and physiological data.

**Analytic target**		**Maximum canonical correlation coefficient**	***P***	**Standardized canonical coefficients of RCS evaluation**				**Standardized canonical coefficients of physiological data**		
				**Being away**	**Extent**	**Fascination**	**Compatibility**	**Pulse rate**	**systolic blood pressure**	**diastolic blood pressure**
Street photographs +S0	RCS	0.172	0.216	–	–	–	–	–	–	–
	Physiological data									
Street photographs +S1	RCS	0.251^**^	0.000	−0.161	−0.711	−0.461	−0.206	0.319	−0.931	−0.029
	Physiological data									
Street photographs +S2	RCS	0.208^*^	0.029	0.692	0.209	0.285	0.309	−0.529	−0.374	0.608
	Physiological data									
Street photographs +S3	RCS	0.282^**^	0.000	1.364	−1.016	0.246	−0.325	0.23	−0.514	1.068
	Physiological data									

* *P < 0.05*

** *P < 0.01*.

## Discussion

### Influence of Streetscape Type on the Subjective Perception Evaluation of Streets in Rural Communities

There were significant differences in participants' VAQ and RCS score for different types of streets. Specifically, the VAQ and RCS scores of artificial–natural enclosed streets and natural semi-enclosed streets were significantly higher than those of other types of streets. Literatures had pointed out that a more visually perceived natural landscape element in the streetscape would bring better physical and mental healing effects (42). The rural cultural imagery and farmland could increase to the rural landscape preference (43). The more obvious the characteristics of the rural environment, the more it can make people feel far away from the daily life of the city (being away), attract people's attention and interest (fascination), and arouse people's rich thinking and association (compatibility) (35). This study further proved that rural community streets with enough greenery, local characteristic structures, and farmland landscape could significantly improve people's visual aesthetic preference and healing perception. Unexpectedly, although artificial buildings were defined by many studies as landscape elements that have negative healing impacts (44), participants rated the artificially enclosed rural community street in this study as highly preferred and perceived it as healing. This may have something to do with the pleasant street aspect ratio (45), because rural communities have much lower building density and height than cities, which makes them visually more open, natural, and attractive, rather than stressful and oppressive.

### Effect of Sound Combinations on the Subjective Perception Evaluation of Streets in Rural Communities

The sound combination had a significant effect on the subjects' visual aesthetic preference, subjective healing perception, and blood pressure. Compared with the silent images, the VAQ and RCS of the subjects were significantly improved, and their blood pressure was significantly reduced when they experienced the audiovisual combinations related to S1. Participants reported significantly better healing sensations when stimulated by nature-related sounds rather than silent images of urban green spaces (9). This study went a step further and found that even in the presence of traffic noise, louder birdsong could significantly improve the healing properties of streets in rural communities. However, when the proportion of bird song decreased to 50 and 30%, the VAQ and RCS score of the scene presented by the same image gradually decreased below the scores of participants observing the silent image. The masking effect of sound should explain this conclusion. Hao et al. demonstrated that bird song does indeed have a masking effect on road traffic noise (32) and can distract people from the type of sound (46). However, this masking effect is significantly reduced when the noise is louder and people's attention is again focused on sound type. Noise often makes people anxious or irritable, which counteracts the therapeutic effects of the environment. While birdsong seems to provide a positive healing experience in either space (31), and traffic noise is the unmistakable “ambience spoiler,” when the two become a composite sound, the situation must be discussed separately. Through quantitative empirical study, this study indicated that adding low-volume birdsong (50% or less) has no significant effect on the healing potential of rural community streets and did not improve the noise environment.

### The VAQ, TR and Evaluation of RCS

The population's aesthetic preferences play a crucial role in the relaxation and healing experience (47). Previous studies have pointed out that visual aesthetics should not simply be associated with the healing effect of the environment (48), but audiovisual perception elements should also be considered as important restorative characteristics of aesthetic value (9). This study proved that there was a positive correlation between the healing perception evaluation and aesthetic preference evaluation of different audiovisual combinations in rural community streets. Moreover, with the increase of the proportion of birdsong in the sound stimulus, participants' healing perception evaluation and aesthetic preference evaluation of the same street photographs increased, and vice versa.

Green space is said to have a significant positive correlation effect on environmental TR (49), and a good visual environment can reduce the perceived sound level by as much as 10 dB (50). This study demonstrated that subjective auditory loudness was influenced by visual and auditory interaction, and the higher the VAQ of the street, the quieter the sound perceived by people. At the same time, when the proportion of traffic noise in the sound combination reached 70%, the visual environment had no significant difference in the participants' evaluation of subjective loudness, which decreased significantly compared with other sound combinations. This finding might indicate that in future environmental planning and design, we should consciously enhance street beauty degree and avoid artificial noise in rural communities so as to create street spaces with restorative potential.

### Physiological Indicators and Evaluation of RCS

In empirical studies on environmental therapeutic efficacy, physiological index data and subjective evaluation were usually combined as the reference standard of environmental healing effect. For example, in the forest recovery study, VAQ was not significantly correlated with eye tracker indicators but had a significant linear correlation with the tranquility rate (TR) (31). Through the canonical correlation analysis between physiological index data and RCS evaluation, this study pointed out that there was no correlation between physiological index data and the evaluation of the RCS when participants only viewed street photographs. In the case of combined audiovisual stimulation, there was a significant correlation between the physiological index data and evaluation of the RCS. Generally speaking, the greater the proportion of birdsong in the audiovisual combination, the higher the evaluation score of the RCS and the lower the pulse, but the blood pressure showed a non-linear trend, possibly because the factors affecting blood pressure are more complex (such as age, sex, and body mass index) (32). Subsequent studies should investigate different populations in detail.

### Implications and Limitations

Empirical studies using a simulated environment with a combination of photographs and audio proved effective, but participants' responses to the landscapes shown in the photographs may be limited by their imagination. Therefore, we suggest the use of VR technology in future quantitative research on the impact of multisensory experience on mental health. In addition, in the context of China's efforts to develop rural community construction, exploring the spiritual recovery needs and preferences of people from different demographic backgrounds is of irreplaceable importance, and studies on participants across broader social and cultural backgrounds are still needed to confirm the validity of the current findings. Finally, the use of two-voice composite audio might be too ideal for a realistic environment. In future research, different types of sound stimuli could be considered to enrich research results (such as other common rural animal sounds), and the finer sound combinations would be considered. Introducing more sophisticated physiological measurement equipment in future research to detect changes in people's physiological and mental states may also be possible.

## Conclusion

As an important aspect of creating a healthy public environment in rural communities, improving street visual landscape and soundscape quality can improve the healing effect of rural community, thereby bringing great health and wellbeing to people. This study indicated that natural landscape factors, rural cultural imagery, and natural sounds can significantly enhance the healing utility of street spaces in rural communities. The data showed that streets with sufficient natural landscape elements and unique rural cultural intention had a significant positive impact on people's visual aesthetic preference, subjective environmental loudness perception, and healing perception. The therapeutic efficacy of street images in rural communities increased significantly with the addition of compound sound with 70% bird song and then decreased gradually with the decrease of bird song and the increase of traffic noise. Therefore, this study points out that the therapeutic effect of rural community streets can be improved by enhancing the rural cultural intention of streets and planting green plants, improving people's visual aesthetic preference and reducing subjective sound loudness. In addition, the soundscape in the streets of rural communities can be optimized to improve the therapeutic efficacy of the streets of rural communities by increasing the proportion of sound levels of birds with positive therapeutic efficacy. Qualitative and quantitative research results complement each other to improve scientific nature and reliability and may also promote more interesting findings in future research.

In view of China's unique national conditions, the rapid development of urban and rural integration in China, and the background of the new national spatial planning, our study provided new insights into the construction of a healthy public environment in rural communities from the standpoint of audiovisual integration. Therefore, designers of future rural community street spaces should consciously provide bird habitats, form beneficial therapeutic landscapes through site optimization and plant screening; and sound the horn to reduce noise in the street environment, so as to give greater play to the healing effect of the audiovisual experience of rural community street spaces.

## Data Availability Statement

The datasets presented in this study can be found in online repositories. The names of the repository/repositories and accession number(s) can be found in the article/Supplementary Material.

## Ethics Statement

The studies involving human participants were reviewed and approved by local Ethics Committee of the College of Landscape Architecture, Sichuan Agricultural University, China. The patients/participants provided their written informed consent to participate in this study. Written informed consent was obtained from the individual(s) for the publication of any potentially identifiable images or data included in this article.

## Author Contributions

EF: conceptualization, supervision, funding acquisition, writing—original draft, and writing—review and editing. YR: formal analysis, investigation, methodology, writing—original draft, and writing—review and editing. XL and LZ: writing—review and editing. All authors contributed to the article and approved the submitted version.

## Funding

This work was supported by Education Department of Sichuan Province (18ZA0377) and National Natural Science Funds of China (Grant No. 52008278).

## Conflict of Interest

The authors declare that the research was conducted in the absence of any commercial or financial relationships that could be construed as a potential conflict of interest.

## Publisher's Note

All claims expressed in this article are solely those of the authors and do not necessarily represent those of their affiliated organizations, or those of the publisher, the editors and the reviewers. Any product that may be evaluated in this article, or claim that may be made by its manufacturer, is not guaranteed or endorsed by the publisher.
