# Supplementary material for: Research on the Healing Potential of Rural Community Streets From the Perspective of Audiovisual Integration: A Case Study of Four Rural Communities in China
Source: Front Public Health. 2022 Mar 11;10:861072. doi: 10.3389/fpubh.2022.861072 (PMC8961884; doi:10.3389/fpubh.2022.861072)
Supplement: Supplementary file 1 [file Table_1.DOCX]

Supplementary Material

# Restorative components scale used in this study

(Imagine you are in the projected scene, please select a scale for each item according to your perception, 0 = ‘totally disagree’, 4 = ‘totally agree’)

| Dimension | | Description | Scales |
| --- | --- | --- | --- |
| RCS | Being-away(B) | B1 There allows me to temporarily forget the troubles of work and daily life. | 0 1 2 3 4 |
|  | Extent(E) | E1 There are many beautiful associations here. | 0 1 2 3 4 |
|  | Fascination(F) | F1 There's a lot here that appeals to me. | 0 1 2 3 4 |
|  | Compatibility(C) | C1 There gives me the opportunity to do what I love to do. | 0 1 2 3 4 |
| TR | | The environment here makes me feel very quiet. | 0 1 2 3 4 |
| VAQ | | The environment here is beautiful and striking. | 0 1 2 3 4 |
